# Methadone versus other opioids for refractory malignant bone pain: a pilot randomised controlled study

**DOI:** 10.1007/s00520-024-08706-w

**Published:** 2024-07-09

**Authors:** Merlina Sulistio, Alexandra Gorelik, Hoong Jiun Tee, Robert Wojnar, David Kissane, Natasha Michael

**Affiliations:** 1 Cabrini Health, Melbourne, VIC 3144 Australia; 2 Faculty of Medicine, Nursing and Health Sciences, Monash University, Melbourne, VIC Australia; 3 School of Medicine, University of Notre Dame Australia, Fremantle, Australia; 4 Musculoskeletal Health and Wiser Healthcare Units, School of Public Health and Preventive Medicine, Monash University, Melbourne, VIC Australia; 5 Department of Medicine, Royal Melbourne Hospital, University of Melbourne, Melbourne, Australia

**Keywords:** Bone pain, Refractory pain, Opioid, Pain control, Methadone

## Abstract

**Purpose:**

Refractory cancer-induced bone pain (CIBP) affects a patient’s functional capacity and quality of life, but there is limited evidence to guide opioid choice. We assessed the feasibility, tolerability and possible efficacy of methadone rotation (MR) compared to other opioid rotations (OOR) in this cohort.

**Methods:**

Adults with CIBP and worst pain intensity ≥ 4/10 and/or opioid toxicity graded ≥ 2 on the Common Terminology Criteria for Adverse Events were randomised 1:1 to methadone or another opioid rotation. Standardised assessment tools were used at pre-defined study time points up to 14 days.

**Results:**

Of 51 eligible participants, 38 (74.5%) consented, and 29 (76.3%, MR: 14, OOR: 15) completed the fourteen days follow-up post-opioid rotation. Both groups displayed significant reduction in average (MR: *d* =  − 1.2, *p* = 0.003, OOR: *d* =  − 0.8, *p* = 0.015) and worst pain (MR: *d* =  − 0.9, *p* = 0.042, OOR: *d* =  − 0.6, *p* = 0.048) and total pain interference score (MR: *d* =  − 1.1, *p* = 0.042, OOR: *d* =  − 0.7, *p* = 0.007). Oral morphine equivalent daily dose was reduced significantly in MR compared to the OOR group (*d* =  − 0.8, *p* = 0.05). The incidence of opioid-related adverse events following MR was unchanged but lower in the OOR group (*d* = 0.9, 95% CI 0.1,1.7, *p* = 0.022). There were no within-group or between-group differences in satisfaction with analgesia at the end of the study.

**Conclusion:**

This pilot study demonstrated that MR and OOR in patients with refractory CIBP are feasible, safe and acceptable to patients. Appropriately powered multi-centre randomised controlled studies are needed to confirm the efficacy of MR and OOR in this cohort.

**Trial registration:**

ACTRN12621000141842 registered 11 February 2021.

**Supplementary Information:**

The online version contains supplementary material available at 10.1007/s00520-024-08706-w.

## Introduction

Cancer-induced bone pain (CIBP) is a common cancer pain syndrome, with a mixture of inflammatory, nociceptive and neuropathic pain requiring a multimodal approach to analgesia management [1]. Current treatments for CIBP include radiotherapy and radioisotopes, opioids, and co-analgesics, pharmaceuticals with antiresorptive properties, and targeted interventional procedures [2, 3]. Despite radiotherapy being the gold standard treatment for painful bone metastasis, studies estimated that 40% of patients fail to respond and only 30% experience complete pain relief [4]. Furthermore, it could take one to fifteen weeks following radiotherapy before pain reduction is observed [4].

Opioids are the foundation of cancer pain management and should be offered to treat moderate-to-severe cancer pain [5]. However, opioids remain underutilised in CIBP [2], with between 25.8% and 84% of patients with moderate-to-severe metastatic bone pain not receiving a strong opioid [6–8]. When an opioid is prescribed, the combination of background and breakthrough (spontaneous and incident) pain commonly seen in CIBP presents challenges in balancing analgesia and opioid adverse effects [9], with the prevalence of breakthrough cancer pain remaining at 59% [10, 11].

Animal modelling of CIBP has revealed a degree of opioid resistance and involvement of neuropathic pain mechanisms [1]. However, no clear clinical benefit has been noted with the routine use of neuropathic agents [3, 12]. A multi-centre, double-blind, randomised controlled trial (RCT) of pregabalin versus placebo in 233 patients with CIBP showed no statistically significant difference in average pain or pain interference between both groups [13]. Hence, there has been limited translation from laboratory knowledge of CIBP into clinical practice to guide the choice of analgesic treatments, including opioid choice [12].

Refractory cancer pain, whereby standard opioid and/or co-analgesic therapy provides inadequate pain relief and/or patients experience unacceptable analgesic adverse effects, is described in 10–20% of cancer patients [14]. The practice of switching from one opioid molecule to another (opioid rotation) for refractory cancer pain is widely supported in palliative care [14, 15]. Rotation to methadone is commonly considered in the management of refractory cancer pain due to methadone’s antagonistic property at the *N*-methyl-d-aspartate (NMDA) receptor and inhibition of serotonin and noradrenaline reuptake [15, 16].

Our preliminary retrospective study of 94 patients rotated to methadone for refractory CIBP demonstrated a reduction in pain intensity from 5.6 to 2.1, with 70% and 53% of patients achieving a ≥ 30% and ≥ 50% reduction in pain, respectively [17]. Methadone rotation (MR) resulted in a reduction in the mean number of daily breakthrough opioid analgesics, with over 70% of patients requiring an actual lower dose of methadone compared to their calculated daily methadone dose [17]. In this pilot trial, we aimed to assess the feasibility, acceptability, safety and possible efficacy of an MR compared to other opioid rotation (OOR) for patients with refractory CIBP. Changes in worst and average pain intensity, effect on pain interference, satisfaction with pain relief and change in opioid requirements will be reported. The trial was registered with the Australia New Zealand Clinical Trial Registry (ACTRN12621000141842) with a detailed study protocol previously published [18].

## Method

### Design and participants

This pilot, open-label, randomised, controlled trial with two parallel groups was conducted between March 2021 and March 2023 at an 850-bed metropolitan hospital in Melbourne, Australia. Convenience sampling was used to screen patients attending the palliative care and radiotherapy departments against eligibility criteria. Eligible participants were ≥ 18 years old with a cancer diagnosis, an estimated prognosis of ≥ 8 weeks and met the diagnostic criteria for CIBP as defined by The Analgesic, Anesthetic, and Addiction Clinical Trial Translations, Innovations, Opportunities, and Networks-American Pain Society (ACTTION-APS) [19]. Participants were additionally defined as having refractory CIBP if they (a) were established on a strong baseline opioid [20]; (b) had an ongoing *worst* pain score of ≥ 4/10 at CIBP site(s) [21]; and/or (c) demonstrated opioid toxicity, with severity grade of ≥ 2 on the Common Terminology Criteria for Adverse Events (CTCAE) v5.0 (Supplementary information 1) [22]. Participants with pain additional to CIBP were eligible for the study, but all pain assessments pertained to the sites where refractory CIBP arose.

Participants with a corrected QT interval of > 500 ms on an electrocardiogram [23], already on methadone, actively receiving radiotherapy or deemed unsuitable for clinical reasons were excluded. We initially excluded those within a week of completing radiotherapy but removed this exclusion due to recruitment challenges. Six patients were recruited prior to protocol alteration.

### Study procedure and randomisation

Following written consent, participants were randomised in a 1:1 ratio using a computer-generated random number sequence with allocation concealed using sealed envelopes. Participants were enrolled and rotated in the inpatient setting from their existing opioid to racemic methadone or another strong opioid (morphine, oxycodone, or hydromorphone) based on best practice guidelines [24]. Participants and investigators were not blinded to the interventions to facilitate dose titration and mitigate the risk of toxicity. The statistician involved in data analysis was blinded to allocation during data analysis.

### Opioid rotation

Opioid rotation was implemented based on published opioid conversion ratios (Supplementary information 2) [25]. For OOR, clinician investigators determined which opioid (morphine, oxycodone, or hydromorphone) to switch to depending on the participant’s opioid history, allergy profile, hepatic/renal function, and clinician preference, allowing for a 25–50% dose reduction to account for incomplete cross-tolerance [26].

MR was conducted using the rapid conversion stop-and-go method [23]. A daily dose of oral methadone (DDOM) was calculated using variable conversion ratios according to the pre-switch OMEDD, as illustrated in Supplementary information 3 [27–29], taking into account potential medication interactions, opioid tolerance and physiological changes affecting volume distribution [23, 25]. Racemic methadone was administered in three or four divided doses. Methadone dosing and frequency were adjusted to clinical effect and observed toxicity, with dose adjustment limited to ≤ 5 mg/ day [23], aiming for twice or thrice daily dosing on discharge.

Unlimited dosing of immediate-release (IR) and/or rapid-onset opioids (ROO) to manage breakthrough cancer pain was allowed. Titration of co-analgesic medications was restricted during the study period to ensure that the observed changes in pain intensity were attributable only to the study intervention. Adjustments to laxatives and other drugs used to manage opioid adverse effects were permitted. Participants were followed up for 14 days from the initiation of the study intervention using face-to-face or over-the-telephone assessments.

### Study objectives

Feasibility was assessed by recruitment and retention rates. Considering a 20–30% attrition rate in palliative care studies, we aimed for > 70% of participants completing the study procedures by day 14 [30]. Acceptability was assessed by the rate of completed data at each study time point and patient satisfaction with analgesia at baseline, day 7 and day 14 post-opioid rotation.

Safety and tolerability of MR and OOR were evaluated using the CTCAE v.5, with grade 2 adverse effects considered moderate severity. A change in the CTCAE composite score for opioid side effects was calculated to compare safety and tolerability between the two study arms [22].

Clinical outcomes assessed were change in worst and average pain intensity on day 14, effect on pain interference, satisfaction with pain relief and overall change in opioid requirements.

### Data collection and measures

**Fig. 1:**
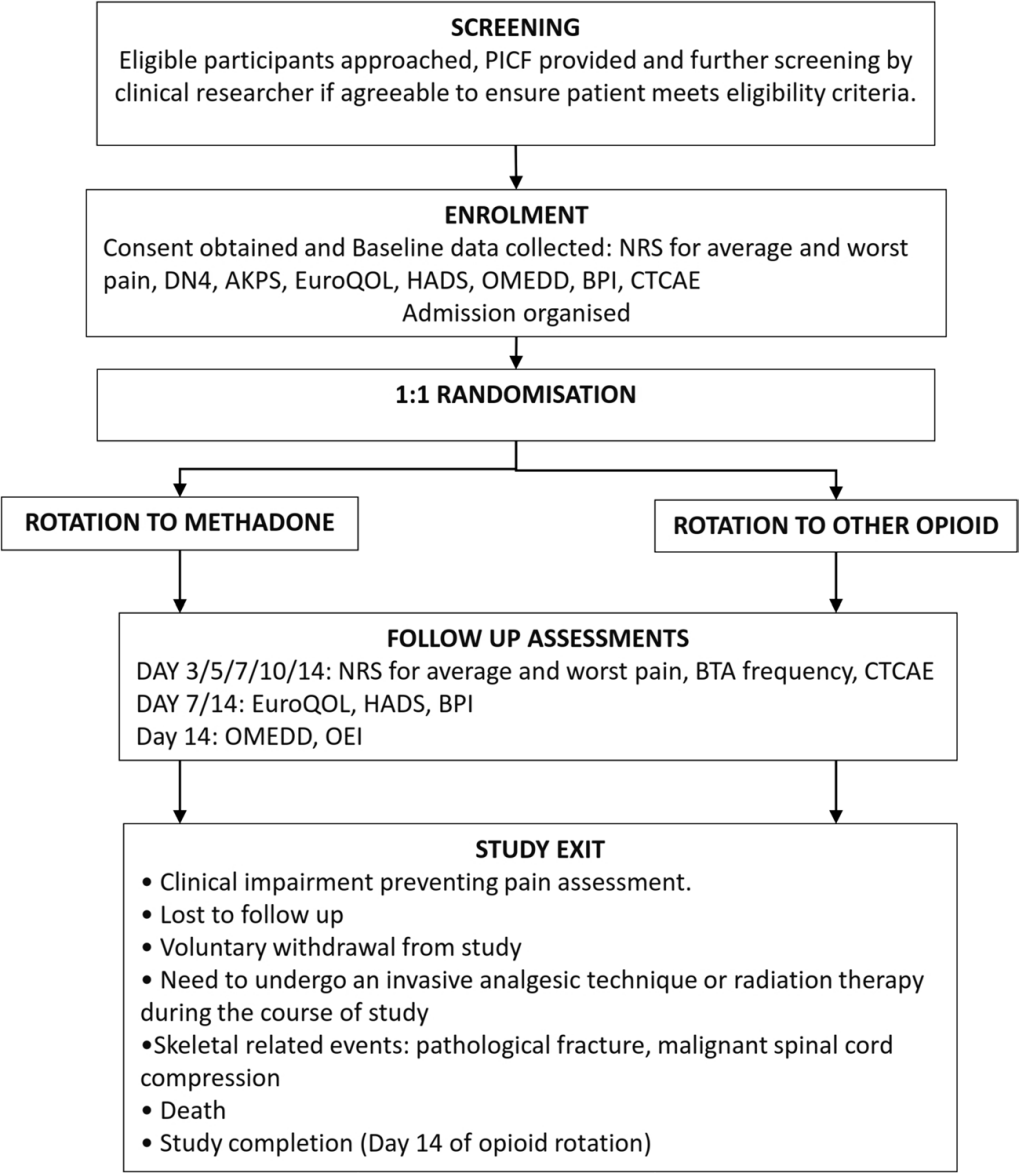
Study procedure. AKPS, Australia-modified Karnofsky Performance Scale; BPI, Brief Pain inventory; CTCAE, Common Terminology Criteria for Adverse Events; DN4, Douleur Neuropathique 4; ECG, electrocardiogram; EuroQOL, quality of life standardised measure; HADS, hospital anxiety and depression scale; NRS, numerical rating scale; OEI, opioid escalation index; OMEDD, oral morphine equivalent daily dose for long-acting opioid analgesia; PICF, patient information and consent form

Participants’ basic demographic and clinical information pertaining to cancer diagnosis, CIBP characteristics and analgesic use were obtained at baseline (Fig. 1). Data were collected at each time point via face-to-face or telephone assessment. The average and worst pain intensities were assessed as per the study procedure (Fig. 1) using a numerical rating score (NRS; 0 – no pain, 10 – most severe pain).

The following standardised assessment tools were utilised as per the study protocol.

- Common Terminology Criteria for Adverse Events: measured opioid adverse events, with a composite score ranging from 0–38 [22].
- Australia-modified Karnofsky Performance Scale: measured performance status [31].
- Douleur Neuropathique 4 (DN4): assessed the neuropathic element of CIBP with a score of ≥ 4/10, suggesting the presence of neuropathic pain [32].
- Subscales of Brief Pain Inventory (BPI): assessed pain interference with daily activities (0 – does not interfere, 10 – complete interference) [33].
- Oral morphine equivalent daily dose (OMEDD): calculated to compare opioid requirements from baseline to end of study (Supplementary information 2).
- Hospital Anxiety and Depression Scale (HADS): screened for anxiety and depression [34].
- EuroQOL thermometer: rated quality of life (QOL) status from 0 (worst) to 100 (best possible) [34].

### Study failure/withdrawal

Participants with severe adverse reactions secondary to MR/OOR, complications unrelated to the study intervention and those who required invasive analgesic techniques or radiation therapy during the study were withdrawn.

### Sample size

With the assumption of a small effect size (0.2) between arms, we aimed to recruit a sample size of 25 per arm, as suggested by Whitehead et al. [35]. Recruitment was affected by the challenges imposed by the coronavirus-19 outbreaks, thus ceased at 38 participants.

### Statistical analysis

The data analysis was performed using the complete case approach. Participants were included in the analysis if they contributed data for both baseline and day 14 assessments. Baseline differences between included and excluded participants were assessed using either the Student T-test or Wilcoxon rank-sum test for continuous data or Fisher’s exact test for categorical variables.

Summary statistics were used to describe study cohort categorical variables and either mean (SD) or median (IQR) for continuous variables. The changes in all outcomes were calculated as a difference between the baseline and the end of the study results. One-sample t-test was used to assess within-group change, while between-group differences were assessed using either the Student T-test or Wilcoxon rank-sum test for continuous data or Chi-square or Fisher’s exact test for categorical variables subject to data distribution and frequencies. Effect sizes were calculated using Cohen’s *d* to provide guidance about the strength of effect given the exploratory nature of this pilot work. Test for proportions was used to examine the between-group differences in the proportion of participants with ≥ 30% and ≥ 50% pain reduction at the end of the study.

Opioid escalation index (OEI%) [36], a surrogate marker of opioid responsiveness and/or opioid tolerance, was calculated using the following equation:

$$\frac{\frac{\text{Total OMEDD at day }14-\text{ Total OMEDD pre}\_\text{rotation}}{\text{Total OMEDD pre}\_\text{rotation}}}{14}*100$$

The oral methadone-to-oral morphine conversion ratio used to calculate OMEDD for participants in the methadone arm at day 14 was 1:4.7 [37]. The data analysis was performed using Stata17 (StataCorp LLC, College Station, TX, USA) with *p* < 0.05 considered statistically significant for all tests. All results have been interpreted with respect to both statistical significance and clinical relevance/importance.

## Results

### Feasibility: participant recruitment, characteristics and retention

**Fig. 2:**
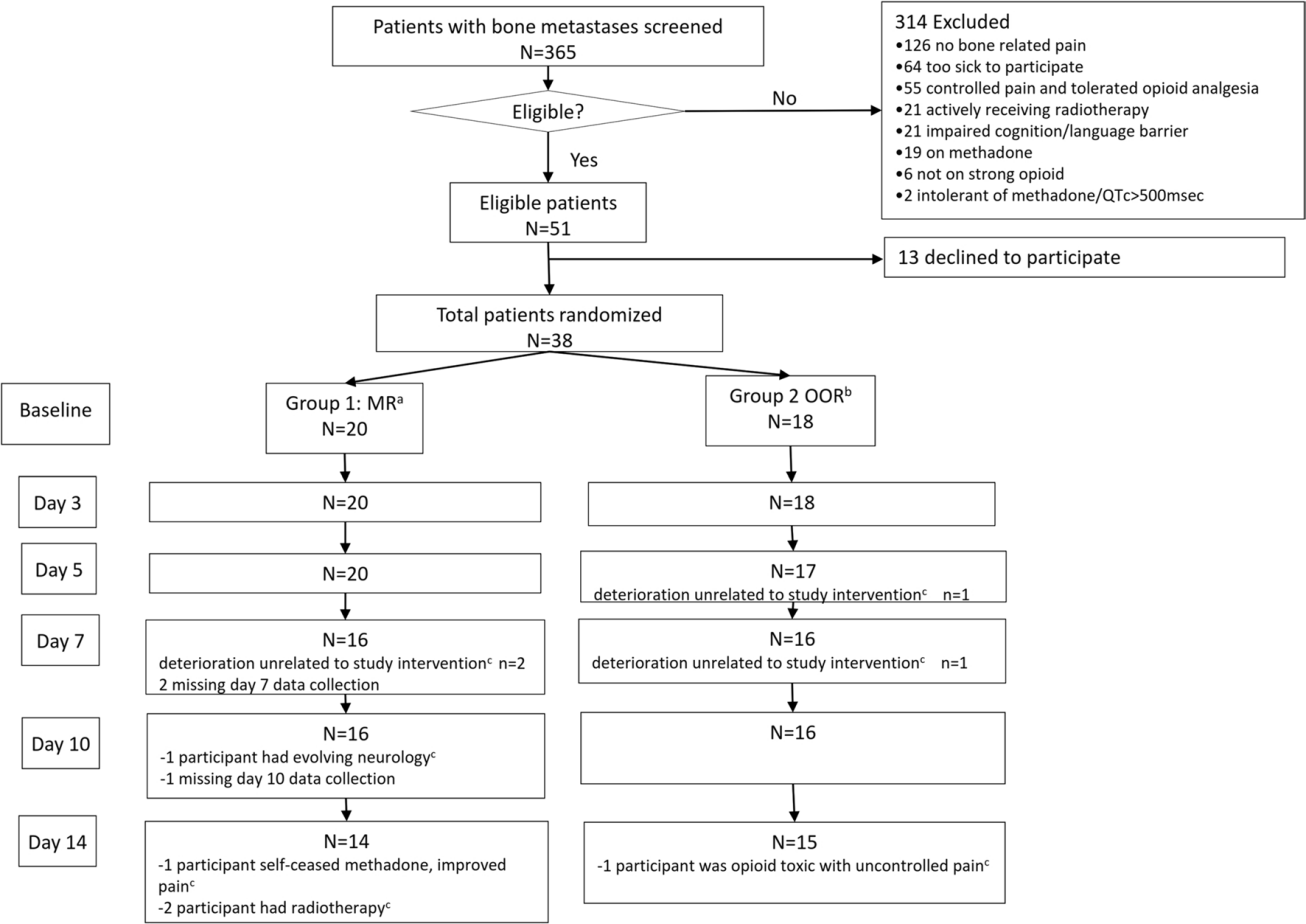
CONSORT (Consolidated Standards of Reporting Trials) participant flow diagram. MR, methadone rotation; OOR, other opioid rotation; QTc, corrected QT interval. ^a^Nine rotated from oxycodone, six from fentanyl, three rotated from morphine and two from hydromorphone. ^b^Eight rotated from fentanyl to hydromorphone, six rotated from morphine to hydromorphone, three rotated from oxycodone to hydromorphone and one rotated from hydromorphone to oxycodone/naloxone. ^c^Withdrawn from study

Figure 2 provides details of screening, randomisation and attrition. Of the 365 patients screened, 51 met the eligibility criteria and 38 (74.5%) consented and were randomised; 20 participants were randomised to MR and 18 to OOR (17 to hydromorphone due to clinician preference), with 14 (70.0%) and 15 (83.3%) participants completing the intervention, respectively (*p* = 0.454). The most common cause for study withdrawal was clinical deterioration unrelated to the intervention.

Table 1 details participants’ baseline demographic and clinical characteristics. Most were female (52.6%), with a mean age of 68 (SD 11.4) years, with a diagnosis of prostate cancer (26.3%) and required occasional care assistance. The spine was the most common site of CIBP (82.9%), with 65.8% of participants reporting multiple sites of CIBP. The median *worse* pain intensity was 8 (IQR 6–9). Half of the participants in the MR group and two-thirds in the OOR group also had ≥ 1 opioid adverse effects graded ≥ 2, with a mean composite CTCAE score of 4 (SD 2). Commonly reported adverse effects were constipation (31.5%) and somnolence (10.5%). The median OMEDD was 85 mg (IQR 60–120), and the mean analgesic satisfaction was 68.1% (SD 22.6).

**Table 1:** Baseline demographic and clinical characteristics

	All patients (N = 38)	MR (n = 20)	OOR (n = 18)	p-value
Demographics				
Age, mean years (SD)	68.3 (11.4)	68.6 (8.3)	68 (14.4)	0.874
Sex, n (%) Female Male	20 (52.6) 18 (47.4)	12 (60.0) 8 (40.0)	8 (44.4) 10 (55.6)	0.338
AKPS, median (IQR)	60 (50–80)	60 (50–80)	70 (50–80)	0.694
Primary diagnosis, n (%)				0.156
Prostate	10 (26.3)	5 (25.0)	5 (27.8)	
Gastrointestinal	6 (15.8)	0	6 (33.3)	
Lung	6 (15.8)	4 (20.0)	2 (11.1)	
Breast	6 (15.8)	4 (20.0)	2 (11.1)	
Renal	2 (5.3)	0	2 (11.1)	
Haematology	2 (5.3)	2 (10.0)	0	
Other^a^	6 (15.8)	5 (25.0)	1 (5.6)	
Site of bone metastases, n (%)^b^				
Spine	35 (92.1)	19 (95.0)	16 (88.9)	0.459
Pelvis	32 (84.2)	17 (85.0)	15 (83.3)	0.616
Rib	21 (55.2)	11 (55.0)	10 (55.6)	0.615
Long bone	20 (52.6)	10 (50.0)	10 (55.6)	0.757
Site of CIBP, n (%)^b^				
Spine	29 (82.9)	14/19 (73.7)	15/16 (93.8)	0.187
Pelvis	22 (68.8)	14/17 (82.4)	8/15 (53.3)	0.128
Long bone	13 (65.0)	6/10 (60.0)	7/10 (70.0)	1.000
Rib	10 (47.6)	5/11 (45.5)	5/10 (50.0)	1.000
Radiotherapy in the last 4 weeks, n (%)	15 (39.5	10 (50.0)	5 (27.8)	0.198
Pain characteristics				
Average pain intensity, median (IQR)	4 (3–6)	5.5 (4–7)	5.5 (4–7)	0.687
Worst pain intensity, median (IQR)	8 (6–9)	8 (7–9)	8 (8–10)	0.209
Neuropathic pain, n (%)	9 (23.7)	5 (25.0)	4 (22.2)	0.604
Total pain interference score, mean (SD)	32.7 (17.9)	36.7 (16.5)	41.1 (16.9)	0.419
Opioids				
OMEDD, median (IQR)	85 (60–120)	90 (60–120)	60 (60–116.3)	0.657
Composite opioid adverse effects score, mean (SD)	4.0 (2.0)	3.6 (2.0)	4.4 (1.8)	0.182
Satisfaction with analgesia, mean (SD)	68.1 (22.6)	63 (23.2)	67.8 (20.2)	0.505
Psychological well being				
HADS, median (IQR) Anxiety Depression	5 (1.5–9) 6 (4–10.5)	3 (1–6) 4.5 (3–7)	6 (3.3–12) 9 (5–12)	0.028 0.029
Quality of life score, mean (SD)	49.4 (22.7)	41.1 (14.3)	51.7 (22.4)	0.087

AKPS, Australia-modified Karnofsky Performance Status; CIBP, cancer-induced bone pain; HADS, hospital anxiety and depression scale; MR, methadone rotation; OMEDD, oral morphine equivalent daily dose; OOR, other opioid rotation

^a^One primary bone cancer in each group; one gynaecological cancer, one cancer of unknown primary and two melanomas in the MR group

^b^% total to greater than 100% as most patients have more than one site of bone metastases/CIBP

The study arms were well balanced, with the only significant difference noted for a higher anxiety and depression score [MR: 3 (IQR 1–6) vs OOR: 6 (IQR 3.3–12), *p* = 0.028, and MR: 4.5 (IQR 3–7) vs OOR: 9 (IQR 5–12), *p* = 0.029, respectively]. Supplementary information 4 details a baseline comparison between study completers and non-completers. The arms in this cohort, too, were well balanced other than for the CTCAE composite score [MR: 3.0 (SD 2) vs OOR: 4.5 (SD 1.9), *p* = 0.041] and anxiety score [MR: 2 (IQR 1–4) vs OOR: 6 (IQR 3–7), *p* = 0.013].

### Acceptability: data completion at each study time point and satisfaction with analgesia

Participants completed, on average, 17 datasets in the MR arm and 16 datasets in the OOR arm across five time points following baseline data collection (Fig. 2). Neither within-group nor between-group differences in satisfaction with analgesia rating reached statistical significance (Table 2).

**Table 2:** Change analysis for all participants who completed 14 days of opioid rotation (*n* = 14 for MR and *n* = 15 for OOR)

	Day 14 measures		Mean change (95% CI)		Within-group effect size(95% CI)		Within-group P-value*		MR/OOR^#^		
	MR	OOR	MR	OOR	MR	OOR	MR	OOR	Mean difference (95% CI)	Effect size (95% CI)	P-value
Pain characteristics (BPI-SF)											
Average pain NRS Median (IQR)	3 (2–4)	4 (3–5)	−2.4 (−3.9, −1.0)	−1.7 (−3.1, −0.4)	−1.2 (−1.9, −0.4)	−0.8 (−1.5, −0.1)	0.003	0.015	−0.7 (−2.6, 1.2)	−0.3 (−1.0, 0.5)	0.458
Worst pain NRS Median (IQR)	5.5 (3–8)	8 (5–8)	−1.8 (−3.5, −0.1)	−1.5 (−2.9, −0.01)	−0.9 (−1.6, −0.2)	−0.6 (−1.3, 0.1)	0.042	0.048	−0.3 (−2.4, 1.8)	−0.1 (−0.8, 0.6)	0.761
Total pain interference Mean (SD)	19.4 (16.0)	30.4 (13.1)	−12.8 (−25.1, −0.5)	−10.9 (−18.3, −3.4)	−1.1 (−1.8, −0.3)	−0.7 (−1.4, 0.001)	0.042	0.007	−1.9 (−15.4, 11.5)	−0.1 (−0.8, 0.6)	0.772
Opioids											
OMEDD Median (IQR)	47 (41.1−–70.5)	80 (40–120)	−36.1 (−63.1, −9.1)	−1.3 (−25.9, 23.3)	0.9 (0.2, 1.7)	0.03 (−0.7, 0.7)	0.013	0.909	−34.8 (−69.5, −0.03)	−0.8 (−1.5, −0.001)	0.050
Escalation index Median (IQR)	−2.9 (−3.8 to −0.1)	−1.2 (−2.4 to 7.1)								−0.8 (−1.5, −0.001)	0.141
CTCAE composite score Mean (SD)	3.4 (2.2)	2.7 (1.8)	0.4 (−0.7, 1.6)	−1.9 (−3.5, −0.2)	−0.1 (−0.8, 0.6)	−1.0 (−1.7, −0.3)	0.426	0.029	2.3 (0.4, 4.2)	0.9 (0.1, 1.7)	0.022
Satisfaction with analgesia Mean (SD)	72.5 (27.1)	69.3 (20.5)	8.2 (−8.6, 25.1)	−0.7 (−8.9, 7.6)	0.4 (−0.3, 1.1)	−0.1 (−0.8, 0.6)	0.312	0.306	8.9 (−8.6, 26.4)	0.4 (−0.4, 1.1)	0.306
Psychological wellbeing											
HADS-Anxiety Median (IQR)	3.5 (1–7)	5 (1–6)	0.8 (−0.6, 2.2)	−1.3 (−2.8, 0.3)	−0.03 (−0.7, 0.7)	−0.4 (−1.1, 0.3)	0.258	0.095	2.1 (0.1, 4.1)	0.8 (0.02, 1.6)	0.043
HADS-Depression Median (IQR)	5.5 (1–9)	6.5 (4–10)	0 (−1.8, 1.8)	−0.3 (−2.8, 2.2)	0.1 (−0.6, 0.7)	−0.2 (−0.9, 0.5)	1.000	0.808	0.3 (−1.6, 1.3)	−0.1 (−0.8, 0.7)	0.842
Quality of life Mean (SD)	42.3 (23.2)	55 (20.6)	3.4 (−8.9, 15.7)	5 (−6.2, 16.2)	−0.1 (−0.8, 0.6)	−0.2 (−0.8, 0.5)	0.560	0.356	−1.6 (17.4, 14.2)	−0.1 (−0.8, 0.7)	0.835

BTA, average frequency of breakthrough analgesia used in 24 h; BPI-SF, brief pain inventory – short form; CTCAE, common terminology criteria for adverse events; HADS, hospital anxiety and depression scale; MR, methadone rotation; NRS, numerical rating scale; OMEDD, oral morphine equivalent daily dose; OOR, other opioid rotation

*The p-value is based on one-sample t-test testing if the change equals 0

^#^Difference between groups in change from the baseline

### Safety and tolerability

Table 3 details the number of reported grade ≥ 2 adverse events and the number of affected participants in the study groups. As illustrated, in most participants, these adverse events improved or resolved by the end of the study, with only 10 participants (6 MR, 4 OOR participants) with grade 2 adverse events (constipation, somnolence, dry mouth and nausea) and no reported grade 3 adverse events at the end of the study. The mean CTCAE composite scores at the end of the study were 3.4 (SD 2.2) for the MR group and 2.7 (SD 1.8) for the OOR group. Within the OOR group, there was a significant reduction in the CTCAE composite score on day 14, with a mean group difference of 2.3 (0.4 to 4.2), *d* = 0.9, *p* = 0.022. Of note, one participant in the OOR group was withdrawn from the study on day 10 due to poorly controlled pain and dose-limiting toxicity.

**Table 3:** Total number of grade 2 or greater adverse events as reported by participants at five pre-defined time points, excluding baseline

CTCAE variables	Grade^a^	MR (*N* = 14)	OOR (*N* = 15)
Constipation	2	8 events, 6 participants (3^b^, 2^c^)	10 events, 6 participants (2^bc^)
	3	0	1
Somnolence	2	7 events, 6 participants (1^b^, 2^c^)	9 events, 7 participants (3^b^, 1^c^)
	3	0	1
Xerostomia	2	5 events, 4 participants (2^bc^)	7 events, 6 participants (2^b^)
Nausea	2	3 events, 2 participants (1^bc^)	3 events, 1 participant^c^
Pruritus	2	3 events, 1 participant^b^	0
Vomiting	2	0	1
Confusion	2	1	0
Hallucinations	2	1	0

CTCAE, Common Terminology Criteria for Adverse Events; MR, methadone rotation; OOR, other opioid rotation

^a^Grade 2 – moderate severity requiring local or non-invasive intervention, limiting the age-appropriate instrumental activity of daily living. Grade 3 – severe or medically significant events requiring hospitalisation or prolongation of hospitalisation, impacting self-care but not life-threatening

^b^Number of participants with event present from screening/baseline

^c^Number of participants with event present at the end of study (day 14)

### Intervention outcomes

#### Pain characteristics

**Fig. 3:**
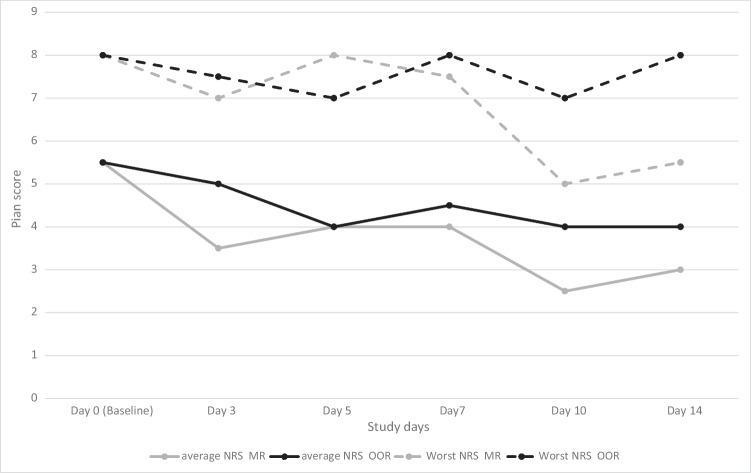
Changes in pain intensity over time. MR, methadone rotation; NRS, numerical rating score; OOR, other opioid rotation

Table 2 shows the significant within-group reduction in average [MR: *d* =  − 1.2 (95% CI − 1.9 to − 0.4), *p* = 0.003 vs. OOR: *d* =  − 0.8 (95% CI − 1.5 to − 0.1), *p* = 0.015] and worst [MR: *d* =  − 0.9 (95% CI − 1.6 to − 0.2), *p* = 0.042 vs. OOR: *d* =  − 0.6 (95% CI − 1.3 to 0.1), *p* = 0.048] pain intensities, with no statistical significance between groups [*d* =  − 0.3 (95% CI − 1.0 to 0.5), *p* = 0.458, for average pain intensity and *d* =  − 0.1 (95% CI − 0.8 to 0.6), *p* = 0.761 for worst pain intensity]. At least a 30% reduction in average pain intensity was observed in 10 participants on methadone (71.4%; 95% CI 47.7–95.1) vs eight participants in the OOR group (53.3%; 95% CI 28.1–78.5%), a mean group difference of 18.1 (95% CI − 16.5 to 52.7, *p* = 0.32). Similarly, at least a 50% reduction in average pain intensity was observed in eight participants on methadone (57.1%; 95% CI 31.2–83.0%) and four participants in the OOR group (26.7%; 95% CI 4.3–64.7%), a mean group difference of 30.4 (95% CI − 3.9 to 64.7, *p* = 0.097). The proportions of responders were less when the worst pain intensity was assessed [MR: 35.7% with at least 30% pain reduction and 28.6% with at least 50% pain reduction vs OOR: 26.7% and 6.7%, *p* = 0.7 and *p* = 0.169, respectively). Whilst both groups displayed improvement in average and worst pain intensities by day 3, the MR group appeared to benefit from further reduction in pain intensities up to day 14 (Fig. 3). Both MR and OOR participants demonstrated a significant reduction in total pain interference [MR: *d* =  − 1.1 (95% CI − 1.8 to − 0.3), *p* = 0.0420; OOR: *d* =  − 0.7, (95% CI − 1.4 to 0.001), *p* = 0.007] with no significant between-group differences (*p* = 0.772).

#### Opioid requirements

OMEDD following MR reduced significantly compared to OOR [*d* =  − 0.8 (95% CI − 1.5 to − 0.001), *p* = 0.05], but there was no difference in the opioid escalation index between groups (*p* = 0.141).

#### Anxiety, depression and QOL

There were no significant differences between arms in participants’ HADS-Depression (*p* = 0.842) or quality of life scores (*p* = 0.835) at the end of the study. Participants in the OOR group demonstrated a non-significant reduction in the HADS-Anxiety score at the end of the study, resulting in a significant between-group difference (*d* = 0.8; *p* = 0.043).

## Discussion

This pilot randomised trial was the first reported opioid rotation study for refractory CIBP. We demonstrated study feasibility and acceptability through the enrolment of 38 participants (75% participation), with 29 participants completing the study (76.3% retention) and only three missing data points amongst the 29 study completers. We found that patients with refractory CIBP were willing to participate in a palliative-focused study and accepted randomisation. We encountered recruitment challenges where patients screened were lost to follow-up with the initial eligibility criteria, requiring participants to wait at least a week following completion of radiotherapy before enrolment. Similarly, given radiotherapy is considered the gold standard treatment for CIBP, some eligible patients declined clinical trials with concern of delaying radiotherapy, and two participants dropped out from the methadone group to proceed with radiotherapy 10 days post-opioid rotation. The unexpected challenges imposed by COVID-19 affected this study recruitment with recruitment closed prior to achieving the targeted 50 participants.

This study participants started with a low CTCAE composite score, with no significant increase in score at the end of the study, suggesting that the study interventions were tolerable and safe. The significant reduction in the CTCAE composite score in the OOR group further suggests that opioid rotation can be beneficial in reducing toxicities through improved opioid responsiveness and resultant reduction in OMEDD.

The low baseline CTCAE composite score in this study implies the practice of ‘early’ opioid rotations before the undue escalation of existing opioids to the point of dose-limiting toxicity. Despite this, we noted a significant reduction in the OMEDD post-MR, suggesting improved opioid responsiveness with methadone. In addition to its action on the mu, delta and kappa opioid receptors, incomplete cross-tolerance may result from the methadone antagonistic effect at the NMDA receptor and the inhibition of serotonin and noradrenaline reuptake [16]. These additional properties of methadone are theoretically beneficial in the modulation of neuropathic pain and prevention of chronic pain, although neuropathic pain studies have demonstrated mixed results [38–40]. In this study, we have noted no difference in the prevalence of assessed neuropathic pain between groups to account for the improved opioid responsiveness seen with MR.

We observed early and sustained pain reduction with MR. Methadone’s greater analgesic potency with repeated administration can be explained by its lipophilic property and phased elimination, resulting in a long and variable half-life of 8–120 h [28]. A reduction in the average pain intensity was achieved in most participants in both groups, but a significant reduction in worst pain intensity was only observed in about a third of participants on methadone and a quarter of participants rotated to another opioid. In this study, we chose at least moderate worst pain intensity as an inclusion criterion, as worst pain has been shown to have a higher correlation with most functional interference scores and has been recommended to evaluate response to radiotherapy for bone metastases [41]. Despite the relatively small proportion of participants with significant *worst* pain reduction, both treatment groups demonstrated a statistically significant reduction in worst pain intensity and total pain interference.

Opioid rotation, whether to methadone or another opioid, is beneficial in reducing both average and worst pain intensities and pain interference. Although not adequately powered, this study suggests that methadone rotation may have the added benefit of further reducing overall opioid requirement, providing earlier and more sustained pain reduction over 14 days with no significant worsening of opioid toxicity compared to baseline.

In considering the implementation of MR in routine clinical practice, it is important that clinicians are familiar with its pharmacokinetics and dynamics. In this study, MR was initiated in the inpatient setting with close monitoring at pre-defined intervals over 14 days. Due to the complex pharmacokinetic and pharmacodynamic profile of methadone, we would emphasise the need for ongoing monitoring beyond 14 days in the ambulatory and community palliative care setting. This requires particular attention if the stop-and-go method of rotation is used due to a higher risk of adverse effects, especially in those on high OMEDD pre-switch.

### Study limitations

This study could not exclude the potential impact of concurrent oncology-specific therapy. The generalisability of this study is limited by the small sample size of single-site recruitment, with most participants in the OOR group being rotated to hydromorphone. We are not able to identify any patient variables or pain characteristics to guide the selection of opioids (methadone vs other opioids). Furthermore, we are not able to firmly conclude on the observed difference in opioid toxicity reporting between groups nor the impact of pain perception by anxiety as the observed significant differences in CTCAE composite scores and anxiety scores between groups may be accounted for by their pre-existing baseline group differences.

Given the pilot nature of this study, we chose to conduct a per-protocol analysis to provide a better estimate of the true efficacy of the study interventions and provide guidance on future studies’ sample sizes. Based on the observed small effect size on pain intensities between groups, a future sample of 123 participants in each group will be required for the study to achieve 80% power to detect a small effect size (Cohen’s *d* = 0.2), assuming two-sided α of 0.05. Future studies will need to consider the high attrition rate in this study population, control for anxiety/depression and the impact of radiotherapy or other concurrent oncology-specific therapy. A larger multi-centre study may also explore patient variables and/or pain characteristics that can guide opioid selection in this patient cohort. To enable multi-centre recruitment and minimise the impact of inpatient access, future studies may consider a different method of MR, such as outpatient titration [18]. Studies of outpatient MR using the stop-and-go method have also been proven to be safe [42, 43] for patients without opioid toxicity pre-switch, although pain stabilisation may be achieved after a considerably longer time [44].

## Conclusion

This pilot RCT demonstrates that rotation to methadone or other opioids in patients with refractory CIBP is feasible and acceptable with comparable efficacy. Methadone rotation provided the additional benefit of lower opioid requirements. This study supports the conduct of an appropriately powered multi-centre RCT to examine the impact of methadone versus other opioid rotation for the management of refractory CIBP.

### Supplementary Information

Below is the link to the electronic supplementary material.

Supplementary file1 (DOCX 27 KB)

Supplementary file2 (DOCX 15 KB)

Supplementary file3 (DOCX 15 KB)

Supplementary file4 (DOCX 19 KB)

## Data Availability

The datasets generated during the study will be available upon reasonable request.

## Declarations

### Provenance and peer review

Not commissioned; externally peer-reviewed.
