# Supplementary material for: Methadone versus other opioids for refractory malignant bone pain: a pilot randomised controlled study
Source: Support Care Cancer. 2024 Jul 9;32(8):495. doi: 10.1007/s00520-024-08706-w (PMC11233296; doi:10.1007/s00520-024-08706-w)
Supplement: Supplementary file 2 — Supplementary file2 (DOCX 15 KB) [file 520_2024_8706_MOESM2_ESM.docx]

## Supplementary 2 Opioid Conversion Ratio [20,26]

| ***Opioid and route*** | ***Analgesic ratio*** |
| --- | --- |
| Oral morphine to oral morphine | 1:1 |
| Oral oxycodone to oral morphine | 3:2 |
| Oral hydromorphone to oral morphine | 5:1 |
| Oral methadone to oral morphine [26] | 1:4.7 |
| Transdermal fentanyl to oral morphine^a^ | 1:100 |
| Oral to subcutaneous morphine | 2-3:1 |
| Oral to subcutaneous oxycodone | 1.5-2:1 |
| Oral to subcutaneous hydromorphone | 2-3:1 |
| Transdermal to subcutaneous fentanyl | 1:1 |

^a^ Example: 60mg oral morphine to 25 mcg/h transdermal fentanyl (equivalent to 0.6 mg/24 h).
