# Supplementary material for: Methadone versus other opioids for refractory malignant bone pain: a pilot randomised controlled study
Source: Support Care Cancer. 2024 Jul 9;32(8):495. doi: 10.1007/s00520-024-08706-w (PMC11233296; doi:10.1007/s00520-024-08706-w)
Supplement: Supplementary file 3 — Supplementary file3 (DOCX 15 KB) [file 520_2024_8706_MOESM3_ESM.docx]

## Supplementary 3 Morphine to Methadone Conversion Ratio Guideline [28-30]

| **Oral Morphine daily dose** | **Conversion Ratio: oral morphine to oral methadone** | | |
| --- | --- | --- | --- |
|  | **Ayonrinde and Bridge 2000 [28]** | **Leppert 2009 [29]** | **Soares 2005 [30]** |
| <100mg | 3:1 | 4: 1 | 5: 1 |
| 101-300mg | 5:1 | 6: 1 | 10: 1 |
| 301-600mg | 10:1 | 12: 1 | 12: 1 |
| 601-800mg | 12:1 | 12: 1 | 12: 1 |
| 801-1000mg | 15:1 | 12: 1 | 12: 1 |
| >1001mg | 20:1 | 20:1 |  |
