# Supplementary material for: Methadone versus other opioids for refractory malignant bone pain: a pilot randomised controlled study
Source: Support Care Cancer. 2024 Jul 9;32(8):495. doi: 10.1007/s00520-024-08706-w (PMC11233296; doi:10.1007/s00520-024-08706-w)
Supplement: Supplementary file 4 — Supplementary file4 (DOCX 19 KB) [file 520_2024_8706_MOESM4_ESM.docx]

| **Supplementary 4** – Baseline comparison between study completers and non-completers | | | | | | |
| --- | --- | --- | --- | --- | --- | --- |
|  | Non-completers (n=9) | Completers (n=29) | p-value | MR completers  (n=14) | OOR completers  (n=15) | p-value |
| *Demographics* | | | | | | |
| Age, mean years (SD) | 68.1 (12.2) | 68.4 (11.4) | 0.952 | 69.9 (6.6) | 66.9 (14.7) | 0.489 |
| Sex, n (%)  Female  Male | 5 (55.6)  4 (44.4) | 16 (55.2)  13 (44.8) | 0.709 | 10 (71.4)  4 (28.6) | 6 (40.0)  9 (60.0) | 0.139 |
| AKPS, median (IQR) | 60 (50-80) | 60 (50-80) | 0.724 | 60 (50-80) | 70 (55-70) | 0.875 |
| Radiotherapy in the last 4 weeks, n (%) | 6 (66.7) | 9 (31.0) | 0.115 | 5 (35.7) | 4 (26.7) | 0.700 |
|  |  |  |  |  |  |  |
| *Pain Characteristics* | | | | | | |
| Average NRS, median (IQR) | 5 (4-6) | 6 (4-7) | 0.728 | 5.5 (4-7) | 6 (3-7) | 0.894 |
| Worst NRS, median (IQR) | 8 (7-9) | 8 (7-9) | 0.686 | 7.5 (6-9) | 8 (8-10) | 0.113 |
| Neuropathic pain, n (%) | 4 (44.4) | 5 (17.2) | 0.174 | 2 (14.3) | 3 (20.0) | 1.000 |
| Total pain interference, mean (SD) | 44.8 (15.0) | 36.9 (16.9) | 0.216 | 32.2 (16.9) | 41.3 (16.3) | 0.153 |
|  |  |  |  |  |  |  |
| *Opioids* | | | | | | |
| OMEDD, median (IQR) | 90 (75-111) | 75 (60-120) | 0.614 | 82.5 (60-120) | 75 (60-120) | 0.888 |
| CTCAE composite score, mean (SD) | 4.7 (1.4) | 3.8 (2.0) | 0.241 | 3.0 (2.0) | 4.5 (1.9) | 0.041 |
| Satisfaction with analgesia, mean (SD) | 58.9 (20.9) | 67.2 (21.9) | 0.319 | 64.3 (25.9) | 70.0 (17.7) | 0.244 |
|  |  |  |  |  |  |  |
| *Psychological wellbeing* | | | | | | |
| HADS-A, median (IQR) | 5 (3-12) | 4 (2-7) | 0.379 | 2 (1-4) | 6 (3-7) | 0.013 |
| HADS-D, median (IQR) | 4 (2-13) | 7 (5-9) | 0.582 | 6 (3-7) | 9 (5-11) | 0.072 |
| Quality of Life score, mean (SD) | 51.1 (11.9) | 44.5 (20.8) | 0.373 | 38.6 (16.4) | 50.0 (23.4) | 0.144 |

CTCAE, common terminology criteria for adverse events; HADS, hospital anxiety and depression scale; MR, methadone rotation; NRS, numerical rating scale; OMEDD, oral morphine equivalent daily dose; OOR, other opioid rotation.
